# The Role of Tomato *WRKY* Genes in Plant Responses to Combined Abiotic and Biotic Stresses

**DOI:** 10.3389/fpls.2018.00801

**Published:** 2018-06-13

**Authors:** Yuling Bai, Sri Sunarti, Christos Kissoudis, Richard G. F. Visser, C. G. van der Linden

**Affiliations:** Plant Breeding, Wageningen University & Research, Wageningen, Netherlands

**Keywords:** abiotic stress, biotic stress, combined stresses, disease resistance, effector-triggered immunity (ETI), PAMP-triggered immunity (PTI)

## Abstract

In the field, plants constantly face a plethora of abiotic and biotic stresses that can impart detrimental effects on plants. In response to multiple stresses, plants can rapidly reprogram their transcriptome through a tightly regulated and highly dynamic regulatory network where WRKY transcription factors can act as activators or repressors. WRKY transcription factors have diverse biological functions in plants, but most notably are key players in plant responses to biotic and abiotic stresses. In tomato there are 83 *WRKY* genes identified. Here we review recent progress on functions of these tomato *WRKY* genes and their homologs in other plant species, such as Arabidopsis and rice, with a special focus on their involvement in responses to abiotic and biotic stresses. In particular, we highlight *WRKY* genes that play a role in plant responses to a combination of abiotic and biotic stresses.

## Introduction

WRKY transcription factors (WRKYs) are a large family of transcriptional regulators, which are defined by the highly conserved WRKY domain (the WRKYGQK motif at the end of the N-terminal and a zinc-finger-like motif at the C-terminus) (Rushton et al., 2010). WRKYs are categorized into three groups (Rushton et al., 2010; Rinerson et al., 2015). Group I (with two WRKY domains) and Group II (with one WRKY domain) contain the zinc-finger-like motif C_2_–H_2_ (C–X_4-5_–C–X_22-23_–H–X_1_–H). Group III contains one WRKY domain and a C_2_–HC zinc-finger-like motif (C–X_7_–C–X_23_–H–X_1_–C) (Eulgem et al., 2000). Based on the primary amino acid sequences, Group II can be further divided into three subgroups (Zhang and Wang, 2005).

Through the binding of the WRKY domain to the W-box *cis*-acting element (consensus sequence: (T)(T)TGAC(C/T)) in the promoters of their target genes, WRKYs can act as transcriptional activators or repressors in regulatory cascades (Rushton et al., 2010; Yokotani et al., 2013; Bakshi and Oelmuller, 2014). The functional specificity of WRKYs is defined by many factors including the W-box (Yan et al., 2013), the WRKY domain (Cheng et al., 2015), interactions with other proteins (Brand et al., 2013; Franco-Zorrilla et al., 2014), and post-translational modifications (Lai et al., 2011).

Many WRKYs have been identified in the plant kingdom (**Supplementary Table S1**). Numerous expression and functional studies have given insight in the involvement of WRKYs in different aspects of plant biology (Van Esse et al., 2009; Rushton et al., 2010; Ishihama and Yoshioka, 2012; Hu et al., 2013; Bakshi and Oelmuller, 2014; Yang et al., 2016). Tomato (*Solanum lycopersicum*) has 83 *SlWRKY* genes (Huang et al., 2012; Karkute et al., 2018). This review focuses on tomato *SlWRKY* genes with regard to their roles in plant responses to biotic and abiotic stresses. The nomenclature of the *SlWRKY* genes follows that of Huang et al. (2012) and Karkute et al. (2018). For *SlWRKY* genes that have not been studied in detail yet, we propose potential roles in response to (a)biotic stresses by looking at their homologs in other plant species (**Supplementary Figure S1**). We paid special attention to the role of *WRKY* genes in the complex regulatory process of plant responses to combined stresses.

## Biotic Stress-Related WRKYs

Plants have developed two layers of induced defense responses (Jones and Dangl, 2006), in which WRKYs are shown to function as either positive or negative regulators (e.g., Bakshi and Oelmuller, 2014; Sarris et al., 2015). The first layer, termed PAMP-triggered immunity (PTI), is activated by the recognition between pathogen-associated molecular patterns (PAMPs) and plant’s pattern recognition receptors. Adapted pathogens can express effector proteins to suppress PTI. The second layer [named effector-triggered immunity (ETI)] is triggered by the recognition of pathogen effectors by plant resistance (R) proteins. Plant R proteins usually comprise nucleotide binding-leucine rich repeat (NB-LRR). PTI and ETI induce both local and systemic acquired resistance responses through the production of reactive oxygen species (ROS) and activation of an integrated signaling network including MAP kinases and hormonal signaling pathways (Dodds and Rathjen, 2010). Salicylic acid (SA), jasmonic acid (JA) and ethylene (ET) are the classical immunity-related hormones.

WRKYs are involved in PTI and ETI at different regulatory levels (Bakshi and Oelmuller, 2014). Firstly, WRKYs can interact (in)directly with PAMPs or effector proteins to activate or repress both PTI and ETI. In barley (*Hordeum vulgare*), *HvWRKY1* and *HvWRKY2* were activated by flg22 (a MAMP) and acted as repressors of PTI against the powdery mildew fungus *Blumeria graminis* f.sp. *hordei*. In addition, the fungal effector AVR_A10_ activated a specific association between the R protein MLA10 and *HvWRKY1/2* leading to inactivation of the repressor function of *HvWRKY1/2* (Shen et al., 2007). In Arabidopsis, *AtWRKY18*, *AtWRKY40*, and *AtWRKY60*, homologs of *HvWRKY1* and *HvWRKY2* (Shen et al., 2007), showed redundant function in negatively regulating PTI to *Pseudomonas syringae* (Xu et al., 2006) and the powdery mildew fungus *Golovinomyces orontii* (Shen et al., 2007). Activation of defense-related genes was observed in *wrky18 wrky40* and *wrky18 wrky60* double mutants and the *wrky18 wrky40 wrky60* triple mutants (Xu et al., 2006; Shen et al., 2007). Similarly, the rice (*Oryza sativa*) *OsWRKY62* gene functions as a negative regulator of both PTI and ETI (conferred by the *Xa21* gene) to *Xanthomonas oryzae* (Peng et al., 2008). These WRKYs are members of the WRKY II-a subfamily and the results above suggest that members of this subfamily may have a conserved negative regulatory function in plant defense. However, overexpression of the WRKY II-a subfamily member *OsWRKY71* enhanced resistance to *Xoo* in rice (Liu et al., 2007). Secondly, WRKYs can be regulated by mitogen-activated protein kinases (MAPKs) (Pandey and Somssich, 2009; Ishihama and Yoshioka, 2012). In *Nicotiana benthamiana*, *NtWRKY7, NtWRKY8, NtWRKY9*, and *NtWRKY11*, phosphorylated by pathogen-responsive MAPKs, were able to bind to the W-box in the promoter of the *RBOHB* gene leading to ROS burst (Ishihama and Yoshioka, 2012; Adachi et al., 2015). *AtWRKY33* interacted with MPK4 and MAP kinase 4 substrate 1 (MKS1) (Andreasson et al., 2005). Upon being challenged with *P. syringae* or upon elicitation by the MAMP flg22, *AtWRKY33* was released from this trimeric complex and subsequently bound to the promotor region of Phytoalexin Deficient3 (PAD3) facilitating the synthesis of antimicrobial camalexin (Qiu et al., 2008; Mao et al., 2011; Ishihama and Yoshioka, 2012). Thirdly, WRKYs regulate hormonal signaling pathways. For example, overexpression of *AtWRKY18* and *AtWRKY70* led to induced expression of defense-related genes, including SA-induced *PR1* (Li et al., 2004). The increased susceptibility to *Botrytis cinerea* of the *atwrky33* Arabidopsis mutant was associated with SA-mediated repression of the JA pathway (Birkenbihl et al., 2012). In addition, WRKYs can contribute to plant immunity by modulating small RNAs (smRNAs), by epigenetic mechanisms through histone methylation, as well as by proteasome-mediated degradation and inter-organelle retrograde signaling (Bakshi and Oelmuller, 2014; Phukan et al., 2016).

**FIGURE 1:**
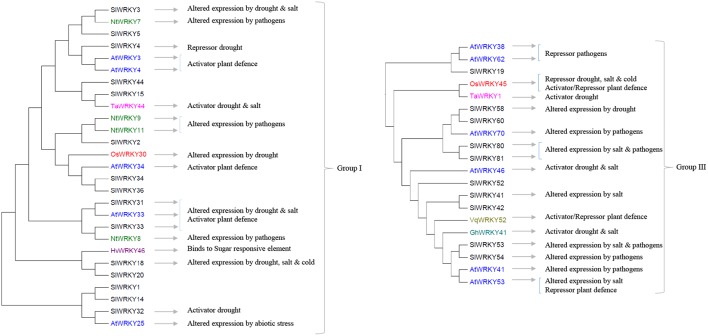
The involvements of Group I and III tomato *SlWRKY* genes and their homologs (highlighted in different colors) in plant responses to biotic and abiotic stresses. The phylogenetic relations of tomato SlWRKYs and their homologs in Arabidopsis (AtWRKYs), rice (OsWRKYs), tobacco (NtWRKY), wheat (TaWRKY), barley (HvWRKY), cotton (GhWRKY), and grape (VqWKRY) are based on the phylogenetic tree presented in **Supplementary Figure S1**.

**FIGURE 2:**
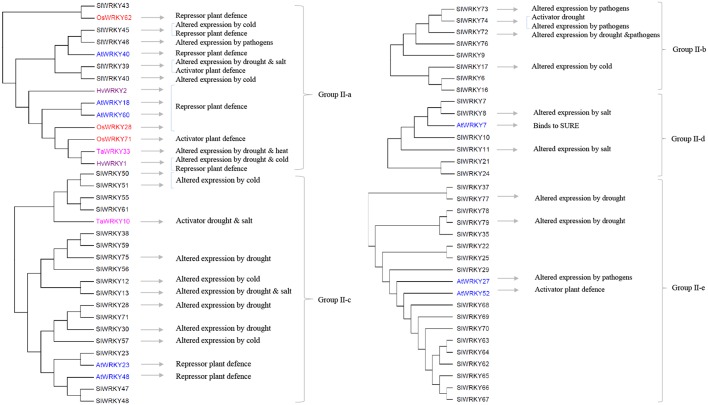
The involvements of the Group II tomato *SlWRKY* genes and their homologs (highlighted in different colors) in plant responses to biotic and abiotic stresses. The phylogenetic relations of tomato SlWRKYs and their homologs in Arabidopsis (AtWRKYs), rice (OsWRKYs), wheat (TaWRKY), and barley (HvWRKY) are based on the phylogenetic tree presented in **Supplementary Figure S1**.

In tomato, WRKYs are studied for their roles in plant defense by either overexpression and/or silencing them (**Supplementary Table S2** and **Figures 1**, **2**). Many tomato WRKYs function as positive regulators of plant responses to biotic stresses. *SlWRKY31* (named *SlDRW1* in Liu et al., 2014) and *SlWRKY33* (named *SlWRKY33B* and *SlWRKY33A* in Zhou et al., 2015), homologs of *AtWRKY33*, were able to complement the compromised tolerance to *B. cinerea* of the *atwrky33* mutant (Zheng et al., 2006). Additionally, overexpression of the *Solanum pimpinellifolium* allele of *SlWRKY33* (named *SpWRKY1* in Li et al., 2015a,b) resulted in resistance to the hemi-biotrophic oomycetes *Phytophthora nicotianae* in tobacco and *Phytophthora infestans* in tomato. The *SlWRKY39* gene, homolog of *AtWRKY40*, was significantly upregulated in tomato upon being challenged with *P. syringae* (Huang et al., 2012) and tomato lines over-expressing *SlWRKY39* showed enhanced resistance to this pathogen (Sun et al., 2015). Overexpression of *SlWRKY45*, another homolog of *AtWRKY40*, enhanced tomato susceptibility to the root-knot nematode *Meloidogyne javanica*, which was associated with decreased expression of JA- and SA marker genes (Chinnapandi et al., 2017). *SlWRKY72*, *SlWRKY73*, or *SlWRKY74* (*SlWRKY72a* or *SlWRKY72b* in Bhattarai et al., 2010) contributed positively to both PTI and *Mi-1*-mediated ETI against root-knot nematodes (*M. javanica*) and potato aphids (*Macrosiphum euphorbiae*) (Bhattarai et al., 2010). Also, *SlWRKY80* (*SlWRKY70* in Atamian et al., 2012) was required for *Mi-1*-mediated resistance against potato aphids and nematodes.

Upon infection of pathogens, altered expression was reported for several tomato WRKYs, including *SlWRKY23* (homolog of *AtWRKY23*), *SlWRKY46* (homolog of *AtWRKY40*), *SlWRKY53/54* (homolog of *AtWRKY23*), *SlWRKY80* and *SlWRKY81* (homologs of *AtWRKY38* and *AtWRKY62*) (Huang et al., 2012, 2016; Du et al., 2015; Lucioli et al., 2016; Rezzonico et al., 2017). Their homologs in Arabidopsis act as negative regulators of plant defense: *AtWRKY38*, *AtWRKY48*, and *AtWRKY62* in the response to *P. syringae* (Xu et al., 2006; Kim et al., 2008; Xing et al., 2008), *AtWRKY23* in response to the nematode *Heterodera schachtii* (Grunewald et al., 2008), and *AtWRKY27* and *AtWRKY53* in response to *Ralstonia solanacearum* (Murray et al., 2007; Mukhtar et al., 2008). Interestingly, overexpression of the grape (*Vitis quinquangularis*) *VqWRKY52* gene in Arabidopsis, a homolog of *AtWRKY53* and *SlWRKY53/54*, enhanced resistance to *Golovinomyces cichoracearum* and *P. syringae*, but increased susceptibility to *B. cinerea*, which was associated with increased expression of SA-pathway related genes and enhanced cell death (Wang et al., 2017). Therefore, further functional analysis of these tomato WRKY genes is needed to confirm their role in either enhanced resistance or increased susceptibility to certain pathogens.

## Abiotic Stress-Related WRKYs

A number of studies demonstrate that WRKYs are involved in plant responses to abiotic stresses, such as drought and salinity (**Supplementary Table S2** and **Figures 1**, **2**). Expression of genes responsive to the signaling hormone ABA was altered in *AtWRKY40* and *AtWRKY40/AtWRKY18* knockout lines. Overexpression of wheat (*Triticum aestivum*) *TaWRKY1* and *TaWRKY33* (a homolog of *AtWRKY40*) in Arabidopsis enhanced drought tolerance through an ABA-dependent pathway (He et al., 2016). The *SlWRKY39* gene, homolog of *AtWRKY40*, was induced by salt, drought, ABA, SA, JA, and *P. syringae* (Huang et al., 2012; Sun et al., 2015). The *SlWRKY45* gene, another homolog of *AtWRKY40*, was upregulated by cold treatment (Chen et al., 2015). *AtWRKY46* was shown to regulate stress tolerance and hormonal response via ABA signaling and auxin homeostasis (Ding et al., 2015).

Overexpression studies of *TaWRKY10* and *TaWRKY44* in tobacco showed that these genes acted as enhancers of drought and salt stress tolerance through regulation of osmotic balance and ROS scavenging (Wang et al., 2013, 2015). Overexpression of the Chrysanthemum *DgWRKY5* gene enhanced tolerance to salt stress by augmenting ROS scavenging and osmotic adjustment (Liang et al., 2017). The rice *OsWRKY30* was involved in drought tolerance in rice via MAPK activation (Rushton et al., 2010; Shen et al., 2012). *DgWRKY5*, *AtWRKY25*, *TaWRKY44*, and *OsWRKY30* are all members of the WRKY family Group I (Liang et al., 2017).

The *AtWRKY46* gene enhances drought and salt stress tolerance, and regulates stomatal closure (Ding et al., 2015). One of its tomato homologs, *SlWRKY41*, was upregulated under salt stress, in addition to *SlWRKY53*, *SlWKRY80*, and *SlWRKY81* (Huang et al., 2012). *SlWRKY58* was upregulated under drought stress (Karkute et al., 2018). Overexpression of the cotton (*Gossypium hirsutum*) *GhWRKY41* gene, the closest homolog of *SlWRKY58*, in tobacco resulted tolerance to drought and salt stress through enhanced stomatal closure as well as by regulating ROS scavenging (Chu et al., 2015).

In addition, altered expression was observed for many other *SlWRKY* genes in tomato, including induction of *SlWRKY23*, *SlWRKY33*, and *SlWRKY57* under salt stress (Huang et al., 2012), upregulation of *SlWRKY12*, *SlWRKY13*, *SlWRKY23*, *SlWRKY50*, and *SlWRKY51* under cold stress (Chen et al., 2015), up-regulated *SlWRKY31* by drought and salt stress (Huang et al., 2012). Under drought stress, *SlWRKY32* and *SlWRKY74* were significantly upregulated (Huang et al., 2012), while *SlWRKY4* was downregulated (Karkute et al., 2015). The possible positive or negative roles of these *SlWKRY* genes in plant responses to abiotic stresses still need to be further verified by functional analyses.

## WRKYs in Crosstalk Between Abiotic- and Biotic-Stress Tolerance

Several of the aforementioned WRKYs are active at crossroads of plant responses to both biotic and abiotic stresses. In Group I (**Figure 1**), *AtWRKY33* and its two tomato homologs *SlWRKY31* and *SlWRKY33* are activators of plant defense to several pathogens (Zheng et al., 2006; Lippok et al., 2007; Liu et al., 2014; Li et al., 2015a). In addition, induction of *SlWRKY31* and *SlWRKY33* was observed under drought and/or salt stresses (Huang et al., 2012). In Group II-a (**Figure 2**), *HvWRKY1* (also designated *HvWRKY38* in Mare et al., 2004), *AtWRKY40* and its tomato homologs *SlWRKY39* and *SlWRKY45* are involved in the response to the infection of pathogens and several abiotic stresses (Xu et al., 2006; Shen et al., 2007; Huang et al., 2012; Chen et al., 2015; Sun et al., 2015; Chinnapandi et al., 2017). Similarly, several WRKYs in Group II-b (**Figure 2**, *SlWRKY72* and *SlWRKY74*) and Group-III (**Figure 1**, *OsWRKY45* and *TaWRKY1*, *SlWRKY80*, and *SlWRKY81*, as well as *SlWRKY53* and *AtWRKY53*) can increase plant tolerance to multiple stresses (Murray et al., 2007; Mukhtar et al., 2008; Qiu and Yu, 2009; Tao et al., 2009, 2011; Bhattarai et al., 2010; Atamian et al., 2012; Huang et al., 2012; Wang et al., 2013, 2015; Marques de Carvalho et al., 2015; He et al., 2016). It is worthwhile to note that WRKYs have been studied for their responses to a single stress at the time. Therefore, further functional analyses of these WRKYs are needed to verify whether the responses to individual stresses remain the same when the plants are exposed to combination(s) of those stress factors. A role for WKRY genes in the interaction of response pathways was obvious in tomato plants in which *SlWRKY23* was silenced (Kissoudis, 2016). These plants exhibited increased resistance to tomato powdery caused by *Oidium neolycopersici*, but this resistance was compromised under salt stress. This example clearly indicates a role for WRKY transcription factors in the crosstalk between biotic and abiotic stress responses, and demonstrates that the responses to individual stresses may not be additive when the plants have to deal with combinatorial stresses.

Tomato is a host for more than 200 species of pathogens, some of which can be controlled by R genes derived from wild tomato relatives (Bai et al., 2018). Evidence is accumulating that plant resistances to pathogens can be attenuated or enhanced by abiotic stresses (Suzuki et al., 2014; Kissoudis et al., 2017). For example, the *Mi-1*-mediated nematode resistance was compromised under heat stress (Marques de Carvalho et al., 2015). Four tomato WRKYs were shown to contribute to the *Mi-1*-mediated nematode resistance [*SlWRKY72* to *SlWRKY74* (Bhattarai et al., 2010) and *SlWRKY80* (Atamian et al., 2012)]. The intriguing question is whether these WRKYs are involved in the instability of the *Mi-1*-mediated resistance under heat stress, or, more generally, do WRKYs play a role in the (in)stability of plant R genes-mediated resistance associated with different molecular mechanisms (Kissoudis et al., 2016).

A (WKRY) gene that confers resistance or tolerance to multiple stresses would be highly useful for breeding. However, *WRKY* genes can also have opposite effects on abiotic and biotic stress tolerance since complex interactions among signaling networks can lead to both synergistic and antagonistic effects on regulation of plant responses to different stresses (Phukan et al., 2016; Bai et al., 2018). For example, *OsWRKY45* that positively mediates broad-spectrum disease resistance while inhibiting adaptation to abiotic stresses (Qiu and Yu, 2009; Tao et al., 2009, 2011), and *OsWRKY75* that increases susceptibility to rice blast fungus while improving tolerance to cold stress (Yokotani et al., 2013). Similarly, other transcription factors have also been shown to play an antagonistic role in modulating responses to abiotic and biotic stresses, such as tomato stress-responsive factor TSRF1 (Zhang et al., 2007), Arabidopsis DEAR1 (DREB (dehydration-responsive element binding protein 1) and EAR (ethylene response factor-associated amphiphilic repression) motif protein 1) (Tsutsui et al., 2009). The regulation of plant responses to multiple stresses relies on tightly regulated and highly dynamic regulatory networks where WRKYs can function as activators or repressors (Eulgem and Somssich, 2007; Bakshi and Oelmuller, 2014; Phukan et al., 2016). Therefore, it is necessary that the roles of WRKYs in a plant’s tolerance to biotic and abiotic stresses should be studied under individual stresses as well as combination(s) of the studied stress factors.

It is important to note that some WRKYs were shown to function in a cluster (Cheng et al., 2015; Phukan et al., 2016), such as the *AtWRKY18-40-60* cluster (Yan et al., 2013). These three WRKYs form both homomeric and heteromeric complexes to modulate downstream target genes and cross-regulate each other, leading to a variety of responses to stresses and during development. It can be difficult to make use of such WRKY-clusters for crop improvement since multiple responses can lead to unwanted traits along with beneficial effects (Phukan et al., 2016). In tomato, five *SlWRKY* genes are close homologs of these three *AtWRKY* genes in Group II-a and shown to be responsive to both abiotic and biotic stresses (**Figure 1**). Further studies are needed to verify whether they also function in clusters and to identify other *SlWRKY* clusters. In this review, we tried to infer functions of unstudied *SlWRKY* genes via their homologs in other plant species. However, it should be stressed that slight changes in the DNA-binding domain may have an important effect on the binding specificity, and sequence homologs may be highly similar yet have different functions (Tao et al., 2009, 2011; Du et al., 2014). For example, the close tomato homologs *SlWKRY3* and *SlWRKY4* are predicted to interact with the W-box DNA through a different motif, RKYGQK, and WRKYGQK, respectively (Lai et al., 2008; Aamir et al., 2017). There is evidence that motifs outside the WRKY domain may provide binding specificity to WRKYs (Phukan et al., 2016). Also, WRKYs have been shown to bind non-W-box elements, including the sugar-responsive element by HvWRKY46, Calmodulin (CaM)-binding domain and the VQ proteins (Phukan et al., 2016). Identification of motifs associated with functions of tomato WRKYs will contribute to the understanding of their regulatory networks under combined stresses.

## Author Contributions

YB designed the outline of the manuscript. YB, SS, and CK contributed to writing and revisions of the manuscript. RV and CvdL contributed to revisions of the manuscript. All authors read and approved the final manuscript.

## Conflict of Interest Statement

The authors declare that the research was conducted in the absence of any commercial or financial relationships that could be construed as a potential conflict of interest.

## Supplementary Material

FIGURE S1 The phylogenetic tree of tomato WRKYs and their homologs in Arabidopsis, rice, tobacco, wheat, barley, and grape. WRKYs of tomato (SlWRKYs), Arabidopsis (AtWRKYs), rice (OsWRKYs), tobacco (NtWRKY), wheat (TaWRKY), barley (HvWRKY), cotton (GhWRKY), and grape (VqWKRY) are colored in black, blue, red, green, fuchsia, purple, teal, and olive, respectively. The evolutionary history was inferred by using the Maximum Likelihood method based on the JTT matrix-based model (Jones et al., 1992) and 500 bootstrap (Felsenstein, 1985). The percentages of bootstrap value higher than 50% are indicated on the nodes.

TABLE S1 WRKY transcription factors discovered in different plant species.

TABLE S2 The involvements of tomato *SlWRKY* genes and their homologs in plant responses to biotic and abiotic stresses.
