# Supplementary figures and images for: The Role of Tomato WRKY Genes in Plant Responses to Combined Abiotic and Biotic Stresses
Source: Front Plant Sci. 2018 Jun 13;9:801. doi: 10.3389/fpls.2018.00801 (PMC6008426; doi:10.3389/fpls.2018.00801)

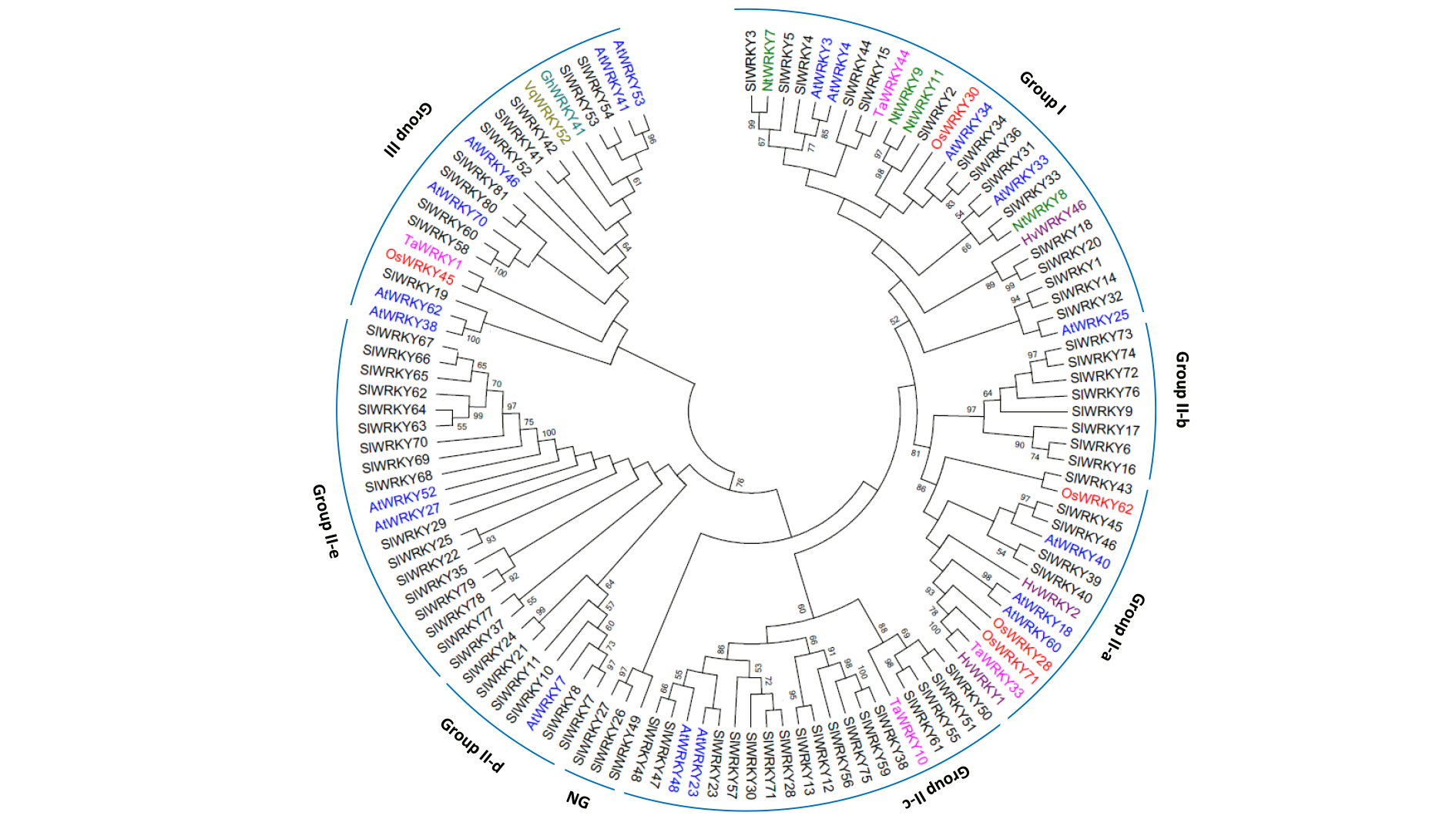

Supplement: FIGURE S1 — The phylogenetic tree of tomato WRKYs and their homologs in Arabidopsis, rice, tobacco, wheat, barley, and grape. WRKYs of tomato (SlWRKYs), Arabidopsis (AtWRKYs), rice (OsWRKYs), tobacco (NtWRKY), wheat (TaWRKY), barley (HvWRKY), cotton (GhWRKY), and grape (VqWKRY) are colored in black, blue, red, green, fuchsia, purple, teal, and olive, respectively. The evolutionary history was inferred by using the Maximum Likelihood method based on the JTT matrix-based model (Jones et al., 1992) and 500 bootstrap (Felsenstein, 1985). The percentages of bootstrap value higher than 50% are indicated on the nodes. [file Image_1.TIF]
