# Supplementary material for: The Role of Tomato WRKY Genes in Plant Responses to Combined Abiotic and Biotic Stresses
Source: Front Plant Sci. 2018 Jun 13;9:801. doi: 10.3389/fpls.2018.00801 (PMC6008426; doi:10.3389/fpls.2018.00801)
Supplement: TABLE S1 — WRKY transcription factors discovered in different plant species. [file Table_1.docx]

Table 1. WRKY transcription factors identified in different plant species.

| Plant species | WRKY Name | Total number | Reference |
| --- | --- | --- | --- |
| Arabidopsis (*Arabidopsis thaliana)* | *AtWRKY* | 74 | (Eulgem, Rushton, Robatzek, & Somssich, 2000; Jixin Dong, Chunhong Chen, 2003; Ülker & Somssich, 2004) |
| Rice (*Oryza sativa japonica)* | *OsWRKY* | 98 | (Ross, Liu, & Shen, 2007; K. Wu et al., 2005) |
| Rice (*Oryza sativa indica)* | *OsWRKY* | 102 | (Ross et al., 2007; K. Wu et al., 2005) |
| Tomato (*Solanum lycopersicum)* | *SlWRKY* | 81 | (Huang et al., 2012) |
| Maize (*Zea mays)* | *ZmWRKY* | 119 | (K.-F. Wei, Chen, Chen, Wu, & Xie, 2012) |
| Grapevine (*Vitis vinifera)* | *VvWRKY* | 59 | (M. Wang et al., 2014) |
| Chinese cabbage (*Brassica rapa)* | *BrWRKY* | 145 | (Kayum et al., 2015; Tang, Wang, Hou, Wang, & Huang, 2014) |
| Wild Foxtail millet (*Setaria viridis)* | *SvWRKY* | 44 | (Muthamilarasan et al., 2015) |
| Foxtail millet (*Setaria italica* (L) P. Beauv) | *SiWRKY* | 105 | (Muthamilarasan et al., 2015) |
| Eggplant (*Solanum melongena* L) | *SmelWRKY* | 50 | (Yang et al., 2015) |
| Turkey Berry (*Solanum torvum* Sw.) | *StorWRKY* | 62 | (Yang et al., 2015) |
| Chinese wild Hazel (*Corylus heterophylla)* | *ChWRKY* | 30 | (Zhao et al., 2015) |
| Wild peanut (*Arachis duranensis)* | *AdWRKY* | 77 | (Song et al., 2016) |
| Wild peanut (*Arachis ipaënsis)* | *AiWRKY* | 75 | (Song et al., 2016) |
| Common bean (*Phaseolus vulgaris)* | *PvWRKY* | 90 | (N. Wang, Xia, & Gao, 2016) |
| Cassava (*Manihot esculenta)* | *MeWRKY* | 85 | (Y. Wei et al., 2016) |
| Oilseed rape (*Brassica napus)* | *BnWRKY* | 287 | (Y. He et al., 2016) |
| Peach (*Prunus persica)* | *PpWRKY* | 58 | (Chen et al., 2016) |
| Carrot (*Daucus carota)* | *DcWRKY* | 95 | (M. Y. Li et al., 2016) |
| Wild Strawberry (*Fragaria vesca*) | *FvWRKY* | 59 | (Zhou et al., 2016) |
| Orchid (*Dendrobium officinale)* | *DoWRKY* | 63 | (C. He et al., 2017) |
| African oil palm (*Elaeis guineensis)* | *EgWRKY* | 95 | (Xiao et al., 2017) |
| Cacao (*Theobroma cacao)* | *TcWRKY* | 61 | (Silva Monteiro de Almeida et al., 2017) |
| Celery (*Apium graveolens L*.) | *AgWRKY* | 69 | (B. Wu, Li, Xu, Wang, & Xiong, 2017) |
| Radish (*Raphanus sativus L.)* | *RsWRKY* | 126 | (Karanja et al., 2017) |
| Sesame (*Sesamum indicum L)*. | *SiWRKYs* | 71 | (D. Li et al., 2017) |
| Potato (*Solanum tuberosum)* | *StWRKY* | 79 | (Zhang et al., 2017) |
